# Taxonomic species recognition should be consistent

**DOI:** 10.1093/nsr/nwad022

**Published:** 2023-01-24

**Authors:** Stephen J O’Brien, Shu-Jin Luo

**Affiliations:** Guy Harvey Oceanographic Center, Halmos College of Arts and Sciences, Nova Southeastern University, USA; The State Key Laboratory of Protein and Plant Gene Research, School of Life Sciences and Peking-Tsinghua Center for Life Sciences, Academy for Advanced Interdisciplinary Studies, Peking University, China

Criteria for species naming have been contentious since the time of Carl Linnaeus. With no clear ‘right or wrong’ convention for species naming, biologists strive to simply convince their peers. This *NSR* compendium offers detailed guidance [1–6] that adds quantitative genomic inference to the cacophony. Certain taxonomic assemblages (e.g. mammals, fish, fungi, insects, terrestrial and marine invertebrates, plants, bacteria) are sufficiently distinctive that any generalization is fraught with natural exceptions.

The biological species concept (BSC), which became the default species definition over 30 different species concepts, defines species as ‘groups of actually or potentially interbreeding populations that are reproductively isolated from other such groups’ [7]. Our opinions derive from decades of molecular genetic research on the ∼40 species of the Felidae and other mammals. The cats enjoy a rich paleontological record, morphometric details, extensive ecology, natural history lore, and a robust molecular phylogeny [8–11]. Combining well-accepted species distinctions (lion-tiger, cheetah-puma, leopard-jaguars, ocelots-margay, each complying with the BSC criteria of reproductive isolation) we compared multiple corelative criteria to specify novel species.

In nominating new species of the clouded leopard, orangutan, elephant and pampas cat, we reported synapomorphic molecular, morphological and cytogenetic traits, distinct nucleotide single nucleotide polymorphisms (SNPs) and microsatellite alleles [12–15]. The new species display reciprocal monophyly, genetic distance corresponding to 1–4 MYA, a high fraction of molecular variance between species, reduced gene flow, and localized hybrid zones. These species identifications have held up with IUCN (the International Union for Conservation of Nature), CITES (the Convention on International Trade in Endangered Species of Wild Fauna and Flora), ESA (the Endangered Species Act), zoo displays and numerous other classifications. Speciation is clearly a dynamic process and achieving reproductively isolated species status in mammals takes time, over a million years for most radiations [16]. With certain caveats, the species status using consistent correlates of the speciation process for these species seems to have been achieved and become widely accepted.

Subspecies is another matter. The subspecies concept itself is controversial. Originally specified by Charles Darwin, subspecies protection has been adopted by the US Fish and Wildlife Service, CITES and IUCN for categorizing endangered species and subspecies needing protection. O’Brien and Mayr defined subspecies as ‘a geographically distinct aggregate of local populations which differ taxonomically from other subdivisions of the species’ [17]. A subspecies has four possible fates: (i) extinction; (ii) connecting and interbreeding with another subspecies to become a new mixed subspecies; (iii) evolution into a new species; and (iv) staying the same [17]. Subspecies are generally distinguished by fixed genetic differences from other subspecies. The key point of designating subspecies is consistency of multiple distinctive criteria, since as for species, there is no right or wrong way to describe subspecies. We further elaborate this point in the Felidae.

Robust genome-wide analyses parsed living tigers into six distinguished subspecies of equivalent genetic distance from each other [18,19]. Yet a proposed revision of Felidae taxonomy ignored the molecular data and lumped all five mainland tiger subspecies into one subspecies, distinct from the Sumatran tiger subspecies [20]. The same revision [20] elevated the Chinese mountain cat, *Felis silvestris bieti*, to a full species designation *Felis bieti*, though it is clearly a subspecies of *F. silvestris* (the wildcat) equidistant from four other *F. silvestris* subspecies such as the Asiatic wildcat *Felis silvestris ornata* [21]. In Southeast Asia, the leopard cat *Prionailurus bengalensis* may reflect a case of ‘failed speciation’, in which two once-diverging Indochinese and Sundaic lineages reconnected, interbred and mixed on today's Malay Peninsula [22]. Therefore, elevating them into two species as proposed [20] seems arbitrary and inconsistent. The inconsistencies here derive from embracing incorrect historical precedents or guesses, and from considering principally morphometric characters while ignoring transformative genomic analyses. Since morphological data are well known to be imprecise, even misleading in phylogenetic considerations [23], these taxonomic mis-classifications are prime examples of ill-informed taxonomic classification due to data denialism.

The lesson here is that consideration of multiple independent measures/correlates of genetic differentiation leads to overall consistency for the taxonomic recognition of species. This is what we personally endorse, particularly in cases where dated fossils, morphology and molecular data are available, more and more the case in today's genomic era.
